# Relaxed and active thin filament structures; a new structural basis for the regulatory mechanism

**DOI:** 10.1016/j.jsb.2017.01.004

**Published:** 2017-03

**Authors:** Danielle M. Paul, John M. Squire, Edward P. Morris

**Affiliations:** a Muscle Contraction Group, School of Physiology, Pharmacology and Neuroscience, University of Bristol, Bristol BS8 1TD, UK; b Division of Structural Biology, The Institute of Cancer Research, London SW3 6JB, UK

**Keywords:** Actin, Tropomyosin, Troponin, Thin filament, Regulation

## Abstract

The structures of muscle thin filaments reconstituted using skeletal actin and cardiac troponin and tropomyosin have been determined with and without bound Ca^2+^ using electron microscopy and reference-free single particle analysis. The resulting density maps have been fitted with atomic models of actin, tropomyosin and troponin showing that: (i) the polarity of the troponin complex is consistent with our 2009 findings, with large shape changes in troponin between the two states; (ii) without Ca^2+^ the tropomyosin pseudo-repeats all lie at almost equivalent positions in the ‘blocked’ position on actin (over subdomains 1 and 2); (iii) in the active state the tropomyosin pseudo-repeats are all displaced towards subdomains 3 and 4 of actin, but the extent of displacement varies within the regulatory unit depending upon the axial location of the pseudo-repeats with respect to troponin. Individual pseudo-repeats with Ca^2+^ bound to troponin can be assigned either to the ‘closed’ state, a partly activated conformation, or the ‘M-state’, a fully activated conformation which has previously been thought to occur only when myosin heads bind. These results lead to a modified view of the steric blocking model of thin filament regulation in which cooperative activation is governed by troponin-mediated local interactions of the pseudo-repeats of tropomyosin with actin.

## 1 Introduction

Contraction in the muscles of vertebrates and some invertebrates is regulated through the thin filaments which contain the proteins actin, tropomyosin and troponin (Brown and Cohen, 2005). Troponin and tropomyosin control the interaction between myosin and actin so that contraction only takes place when the intracellular Ca^2+^ concentration is elevated. Elucidating the roles played by troponin and tropomyosin in this mechanism is essential for a full understanding of thin filament regulation and is of medical interest since mutations in these proteins can lead to inherited cardiomyopathies and other diseases (Tardiff, 2011).

In the thin filament, G-actin monomers polymerize to form the F-actin filament, a helical polymer of actin subunits that appears as two slowly twisting strands. Tropomyosin molecules are dimeric α-helical coiled-coils ∼400 Å in length (Li et al., 2011). linked end-to-end to form two helical strands following the long period helices of F-actin in the thin filament. Each tropomyosin molecule spans seven actin subunits and interacts with one troponin complex. The structure of F-actin in complex with tropomyosin has been determined using cryo electron microscopy with actin at a resolution of 3.7 Å and tropomyosin at a resolution of 6.7 Å (von der Ecken et al., 2014). Comparison with an 8 Å structure of actin-tropomyosin decorated with myosin heads by the same group (Behrmann et al., 2012) has shown a myosin induced transition of tropomyosin: but for a full understanding of regulation the regulatory protein troponin must also be included.

Troponin contains three subunits: TnC the Ca^2+^ binding switch, TnT which binds to tropomyosin and TnI which has an inhibitory role. It comprises an extended tail formed by the N-terminal region of TnT (TnT1; residues 1–158) and a globular domain formed by the rest of TnT (TnT2. together with TnI and TnC. Partial crystal structures exist for the core domain of troponin (TnC, part of TnI and TnT; (Takeda et al., 2003, Vinogradova et al., 2005)). Together, these individual structures define the overall structure of the thin filament provided that their relative geometries and configurations are known. A theoretical model of the full thin filament has been generated from this information with structure prediction tools used for the missing regions (Manning et al., 2011).

X-ray diffraction experiments suggest that muscle activation involves a movement of tropomyosin on actin, leading to the steric blocking model of regulation (Haselgrove and Huxley, 1973, Huxley, 1972, Parry and Squire, 1973). Here tropomyosin blocks the myosin binding sites on actin in relaxed muscle and Ca^2+^ binding to troponin shifts tropomyosin sideways exposing these sites so that myosin can bind. Tropomyosin has been resolved in different positions in electron microscope (EM) reconstructions of thin filaments in the on and off states (Lehman et al., 1994, Lehman et al., 1995). These all used helical reconstruction procedures in which every actin subunit is treated as being identical and the density of tropomyosin and troponin is averaged onto every actin subunit (Squire and Morris, 1998). Single particle procedures that do not assume helical symmetry are needed to properly recover the structure of troponin and the different regions of tropomyosin from EM data (Paul et al., 2004, Paul et al., 2010, Paul et al., 2009).

Tropomyosin movement across the surface of actin revealing the myosin binding sites have been refined into a three state model (Lehrer and Geeves, 1998, McKillop and Geeves, 1993) in which, in the absence of Ca^2+^, tropomyosin lies over the inner edge of the outer actin domains (1 and 2) blocking the myosin binding sites (Blocked or B state). The binding of Ca^2+^ causes tropomyosin to move across to the outer edge of the inner actin domains (3 and 4) allowing restricted myosin binding (Closed or C state); the binding sites are not yet fully available. Subsequent myosin binding causes further movement of tropomyosin, exposing all the binding sites and leading to full activity (Myosin or M-state). Kinetic data suggested that tropomyosin is in equilibrium between the three states (Lehrer and Geeves, 1998, McKillop and Geeves, 1993). Structurally, three regulatory states and three positions of tropomyosin were recorded using helical reconstruction of EM data (Lehman et al., 1994, Vibert et al., 1997, Xu et al., 1999).

The data analysed here have previously been used in a different single particle approach (Pirani et al., 2005) where various references calculated from partial crystal structures of troponin were used to align the EM images. This resulted in the recovery of a troponin core domain which was substantially similar to the original reference with very little movement between the Ca^2+^-treated and Ca^2+^-free states.

## 2 Three-dimensional analysis of relaxed and active thin filaments

**Fig. 1:**
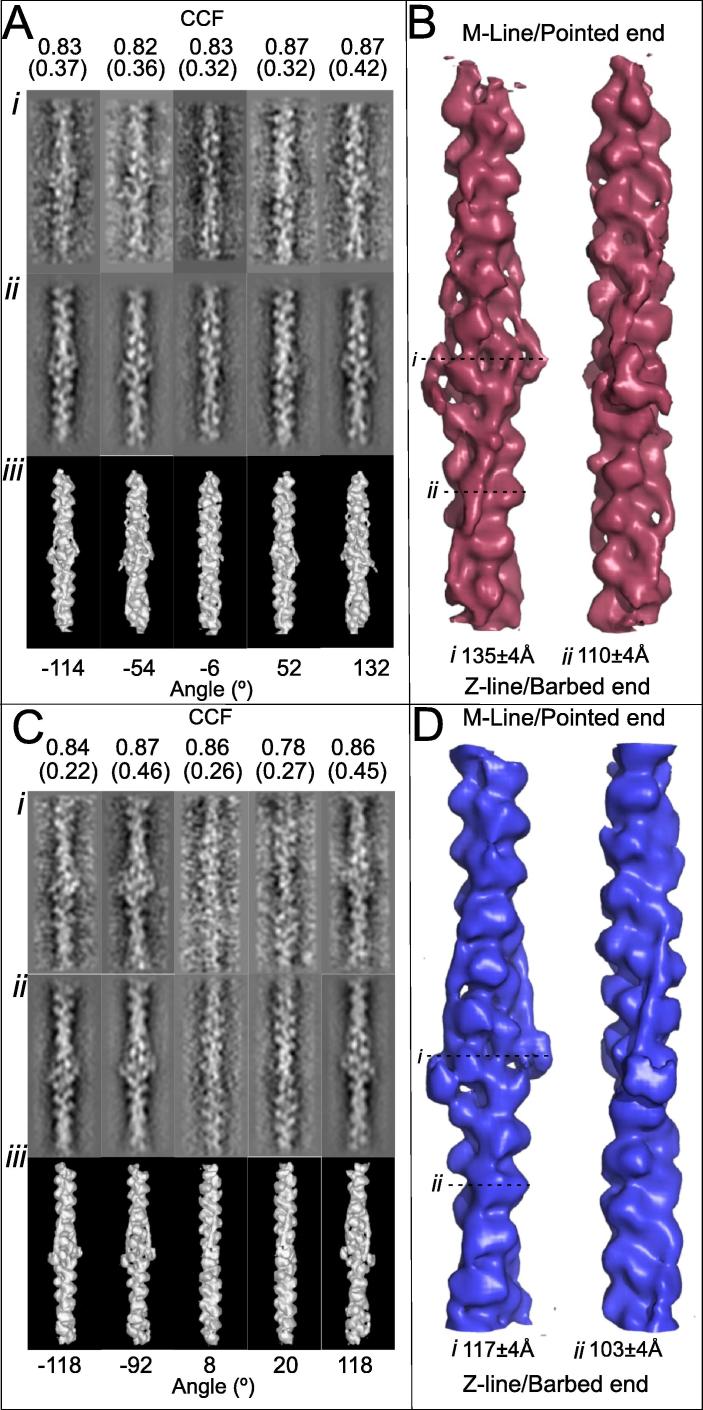
Comparison of data from Ca^2+^-free and Ca^2+^-treated filaments. (A and B) Data from Ca^2+^-treated filaments, (C and D) data from Ca^2+^-free filaments. (A and C) The five columns represent different viewing angles of the filaments described by the Euler angles α = 0°, β = 90° throughout and then with the angles γ shown below each figure. Rows Ai and Ci are class averages, Aii and Cii are reprojections and Aiii and Ciii are surface views of the 3D reconstruction displayed at the corresponding angles. The cross-correlation coefficient between the class averages and the corresponding reprojections are given at the top of each column for the Ca^2+^-treated state (A) and the corresponding cross-correlations between the inverted class average and the reprojection are shown underneath in brackets. Similar cross-correlations are shown in C for the Ca^2+^-free state (C). (B and D) The surface rendered 3D maps of the Ca^2+^-treated filament (B) and the Ca^2+^-free filament (D). Two viewing angles of the reconstructions are shown with a 90° rotation about the filament axis between the two.

Two sets of EM images of negatively stained reconstituted thin filaments were analysed. The first set of 408 filaments had been treated with EGTA to induce the Ca^2+^-free conformation and a second set had 138 Ca^2+^-treated filaments. The filaments were prepared and imaged by Pirani et al. (2005) and subsequently processed for the work described here using single particle-based procedures (Paul et al., 2010, Squire et al., 2017) yielding final structures for the Ca^2+^-free and Ca^2+^-treated thin filaments (Fig. 1). Resolution estimates using Fourier shell correlation (FSC) (Bottcher et al., 1997) were 28.4 Å for the Ca^2+^-free and 27.7 Å for the Ca^2+^-treated structures. The two maps have been deposited in the EMDB with accession numbers 3578 and 3576 respectively). We can readily identify the actin subunit cleft in our maps which becomes visible between 25 Å & 30 Å consistent with these values. In both Ca^2+^- treated and Ca^2+^-free states globular density attributable to troponin is well recovered. In the former, troponin has an open conformation with extensions or arms of density emerging from the central core region (Fig. 1A and B). In the latter (Fig. 1C and D) troponin is more compact and more closely associated with actin. In each case troponin density labels the two strands of the actin filament with a stagger of 27.5 ± 0.5 Å, the axial rise between actin subunits along the F-actin genetic helix and matches our previous measurements (Paul et al., 2009). This similar arrangement in reconstituted thin filaments and in native systems is thus unlikely to be solely a consequence of the assembly process in the sarcomere (as considered previously (Paul et al., 2009)) and may arise from cooperative binding of the troponin-tropomyosin complexes across the two long-pitched strands of the actin filament.

## 3 Docking of actin and troponin/ tropomyosin into the observed density

**Fig. 2:**
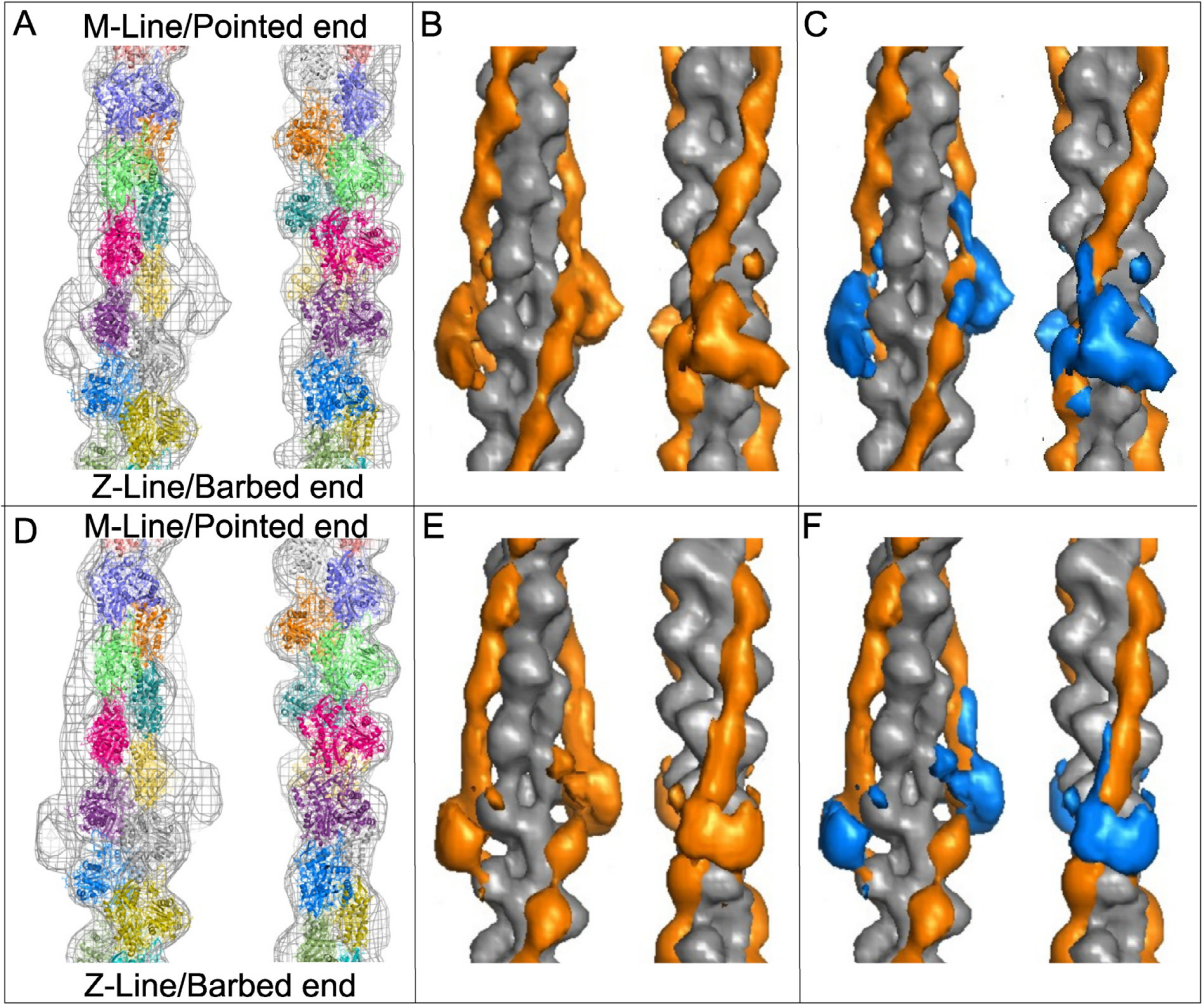
Difference density analysis. (A–C) Data from Ca^2+^-treated filaments, (D–F) data from Ca^2+^-free filaments. (A and D) Wire mesh representation of the single particle based reconstructions of the thin filament in the two states. An atomic F-actin model is docked into the reconstruction and each subunit is colour coded. The barbed end (Z-band end) of the actin filament is at the bottom of the figure. (B and E) Difference density maps calculated by subtracting the docked F-actin model (grey) from the single particle reconstructions. This leaves the density attributable to the regulatory proteins troponin and tropomyosin (both orange). (C and F) Difference density maps calculated by subtracting docked F-actin (grey) and tropomyosin (orange) models from the single particle reconstructions leaving density attributable only to troponin (blue).

To interpret the maps at a molecular level an F-actin atomic model based on the 6.6 Å cryo-EM structure (Fujii et al., 2010) was docked into the 3D density (Fig. 2A and D). The model coordinates have been deposited: PDB: 5MVA corresponds to the high Ca^2+^ map EMDB-3576 and PDB: 5MVY corresponds to the low Ca^2+^ EMDB-3578. Model F-actin densities were calculated from the docked actin backbone and subtracted from the reconstructions to produce difference density maps for the tropomyosin/troponin complexes in the two states (Fig. 2B, E). These reveal extended rod-like densities attributed to tropomyosin, together with more globular troponin densities. The troponin/tropomyosin complex was further dissected by docking atomic models for the tropomyosin strands and subtracting the model density of the actin/tropomyosin backbones to derive the structure of the troponin molecules alone (Fig. 2C and F).

**Fig. 3:**
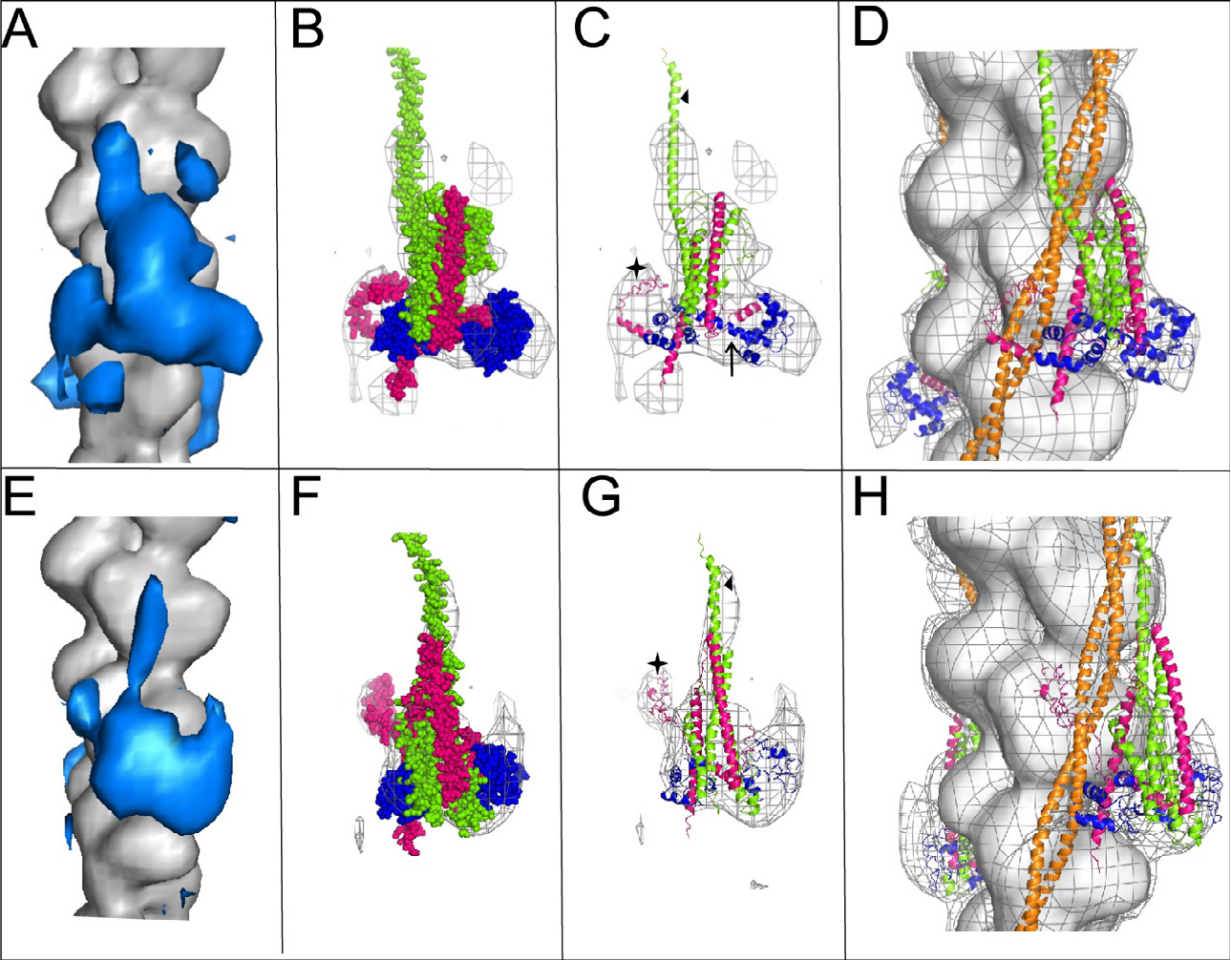
Fitting the troponin density. (A–D) Data from Ca^2+^-treated filaments, (E–H) data from Ca^2+^-free filaments. (A and E) The density attributable to troponin is shown (blue) in the Ca^2+^-treated state (A) and Ca^2+^-free state (E). (B, C and F, G) Space filling (B and F) and ribbon diagrams (C and G) of models of the troponin complex based on the crystal structures of the core domains: 1YVO for the Ca^2+^-free state and 1YVTZ for the Ca^2+^-treated state (Vinogradova et al., 2005) together with the atomistic model of the whole complex (Manning et al., 2011) that returned the best fit docked into the electron density envelopes produced by the difference analysis. The three troponin components are displayed in different colours: TnC, blue; TnT green; TnI pink. (C and G) The location of the C-terminal region of TnI is highlighted by a star, TnT1 is indicated by a triangle and the ordered central helix of TnC is arrowed in C. (D and H) Maps of the Ca^2+^-treated (D) and Ca^2+^-free (H) filaments with all the components docked into the density (tropomyosin shown in orange).

The major domain of the troponin complex in the difference analysis changes quite dramatically from an L-shaped density in the active state (Fig. 3A) to a bi-lobed mass in the Ca^2+^-free state (Fig. 3E), in each case retaining rather similar thin extensions closely associated with tropomyosin which run towards the pointed (M-line) end of the actin filament. These are likely to arise from the elongated sub-domain of troponin-T (troponin T_1_) which binds to tropomyosin; this position of TnT1 is in agreement with our previous observations (Paul et al., 2009). The density attributed to TnT1 spans two actin subunits, a length of ∼110 Å. We have not recovered the expected full length of 180 Å (Flicker et al., 1982).

A detailed interpretation of the troponin domains in the two states was made by quantitative docking of the crystal structures of the troponin core into the troponin densities. All of the crystal structures of the troponin complex currently available are incomplete. The most comprehensive structure comprises ∼60% of the total representing the troponin core (Takeda et al., 2003, Vinogradova et al., 2005). The rest of the troponin complex has been predicted in an atomic model of the whole troponin complex (Manning et al., 2011). To establish which model and orientation gave the best fit to our protein density we docked a set of six models for the Ca^2+^-treated state and four for the Ca^2+^-free state. For both the Ca^2+^-treated and Ca^2+^-free thin filament reconstructions the best agreement was observed with a composite model based on the crystal structures of the core domain of skeletal troponin in the relevant Ca^2+^ binding state (1YTZ and 1YVO; (Vinogradova et al., 2005)) in which the missing regions of the full troponin complex were built in using the predicted model (Manning et al., 2011). The composite models are oriented so that the base of the molecule formed from the two lobes of TnC points towards the barbed end of the thin filament, while the apex formed from the Tn I α-helices is oriented towards the pointed or M-band end of the actin filament (Fig. 3B–D and E–H). The crystal structures 1YTZ and 1YV0 on their own returned the next best fit in a similar orientation. None of the trial models accounted for all the density recovered in our EM maps in particular around the N-lobe of TnC. In each case the best fitting orientation found for the troponin molecules on the thin filament is similar to that previously observed by us (Paul et al., 2009). It is approximately opposite to that described by the Lehman group (Pirani et al., 2005, Yang et al., 2014) and *in situ* protein domain orientation experiments (Sevrieva et al., 2014). Interatomic distances in the docked models of the Ca^2+^-free and Ca^2+^-treated states can be compared with published FRET distance measurements for residues in troponin I and actin (Kobayashi et al., 2001, Kobayashi et al., 2000, Li et al., 2001). The overall measurements agree with the reported FRET measurements when taking into account the probe size. The movement that we see between the two states is larger (13 Å) than the reported (6–8 Å) for the more mobile region of TnI117 whereas TnI96 which sits in the IT coiled-coil arm moves very little (3.3 Å) which agrees with the reported values (3.6 Å).

## 4 Movement of tropomyosin

**Fig. 4:**
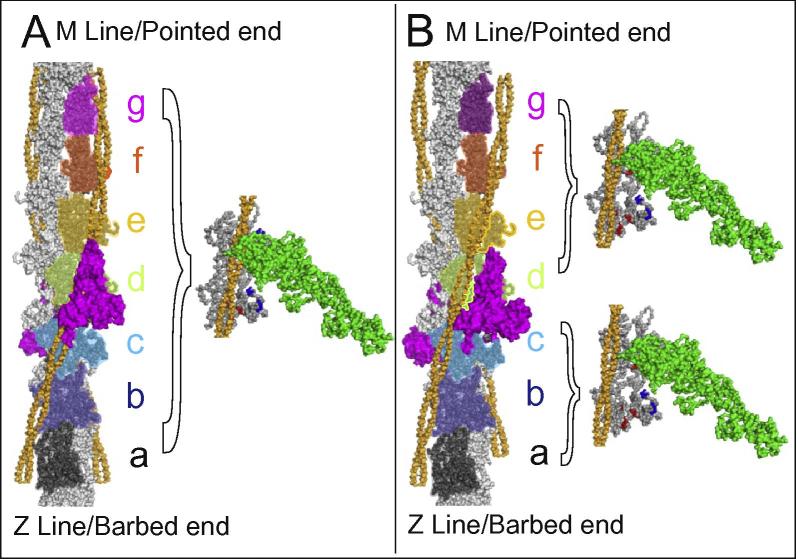
Visualisation of the tropomyosin domain movements and myosin binding. (A) Structures of the Ca^2+^-free state and (B) structures of the Ca^2+^-treated thin filament assembly. (A and B) show the central region of the Ca^2+^-free (A) and the Ca^2+^-treated (B) reconstructions over a length of 14 actin subunits, two strands of tropomyosin (orange) and two troponin complexes. The core domains of the COMPHI model are in magenta. Subunits a to g of one strand of actin are colour coded and the second strand is grey. In the Ca^2+^-free state (A) the position of tropomyosin is the same on every subunit and lies in the B or blocked position where it would inhibit myosin binding. The position of tropomyosin is different on different subunits of the actin filament in the Ca^2+^-treated state (B), where the average positions are illustrated for actin subunits d to g (closed state) and a to c (M-state). A & B (right inserts) show face on views of the actin subunits, with weak (blue) and strong (red) actin binding sites highlighted for the three distinct positions of tropomyosin. Subunits a, b and c show tropomyosin in the “M or Myosin state”, subunits d, e, f, g show tropomyosin in a position more closely aligned to the “C or closed” state. In the Ca^2+^-treated thin filament a myosin S1 head (green) can access all weak and potentially some strong binding sites on subunits e, f and g and presumably all available binding sites on subunits a and b. An equivalent myosin head would be blocked on every subunit in the Ca^2+^-free state.

Docking of tropomyosin strands into the difference maps of the thin filament highlighted the significant difference in their position relative to actin that is associated with the transition from the Ca^2+^-free to the Ca^2+^-treated state. Tropomyosin moves across each actin subunit from the inner edge of the outer domain of actin in the Ca^2+^-free state (Fig.4A) to the outer aspect of the inner domain in the Ca^2+^-treated state (Fig. 4B). This general movement is similar to that previously described from helical thin filament reconstructions (Lehman et al., 1995, Vibert et al., 1997, Xu et al., 1999), and forms the basis for current understanding of the steric mechanism of regulation of the thin filament. However, the single particle approach used in the current study yields independent structures for each actin subunit and its associated tropomyosin pseudo-repeat and has revealed marked differences in the positions of these pseudo-repeats on different actin subunits in the Ca^2+^-treated state. This prompted us to study the exact position of tropomyosin with respect to each actin subunit for both the Ca^2+^-treated and Ca^2+^-free states.

The exact location and movement of tropomyosin was investigated through docking experiments using an atomic model of tropomyosin (Orzechowski et al., 2014) in a three step procedure. The best fit for the entire length of the reconstruction was calculated first. This ‘full length’ fit was then refined to allow more localised fitting in which each subunit along a strand was considered in turn and the best position was found for the segment of tropomyosin spanning the chosen actin subunit as well as the subunits on either side along the strand (i.e. a run of three actin subunits). The analysis focused on the central region of our reconstruction, a length comprising fourteen actin subunits, two strands of tropomyosin and two troponin complexes, corresponding to the functional unit of the system (Fig. 4A and B). To fit the position of tropomyosin correctly at the ends of the functional unit extra actin subunits above and below were also considered. Finally molecular dynamics flexible fitting software ‘mdff’ (Trabuco et al., 2008) was used to check and refine the manual fit and URO results. The tropomyosin strands in the Ca^2+^-free state (Fig. 4A) were found to lie in an almost identical position with respect to their neighbouring actin subunits, showing very little variation in radius or azimuth and being located close to the inner edge of the actin outer subdomains (1 and 2). This position has previously been termed the blocked position, since the majority of myosin binding sites are obscured and access to the strong binding sites of myosin is completely blocked (Lehman et al., 1995). This differs from a previous single particle-based analysis in which it was proposed that in the Ca^2+^-free state of the thin filament the path of the tropomyosin strands is deflected by troponin (Murakami et al., 2005).

In contrast to the rather similar tropomyosin positions for each regulatory unit in the Ca^2+^ free state, in the Ca^2+^-treated state, the position of tropomyosin relative to actin varies systematically along the thin filament (Fig. 4B). The individual positions of the tropomyosin pseudo-repeats can be divided broadly into two groups identified by their adjacent actin subunits. To aid identification, the actin subunits are labelled a–g in Fig. 4. Subunits c and d are adjacent to the troponin complex, while subunits a and b are located towards the barbed end of thin filament and subunits e, f and g are located towards the pointed end. Tropomyosin strands adjacent to actin subunits d, e, f and g show the least displacement compared to the Ca^2+^-free state, corresponding to a mean rotation around the thin filament axis of 18°. This places these tropomyosin repeats over the outer aspect of the inner subdomains (3 and 4) of the actin subunits (Fig. 4B) and corresponds quite closely to the conformation previously described for the Ca^2+^-treated state of the thin filament which has been termed the Closed or C-state of tropomyosin (Lehman et al., 1995, Vibert et al., 1997, Xu et al., 1999). In comparison, tropomyosin repeats adjacent to actin subunits a, b and c show a significantly larger displacement compared to the Ca^2+^-free state with a mean rotation of 28°. Here the tropomyosin strands are located over the inner edge of the inner domains of the actin subunits (Fig. 4B) so that the myosin binding sites are now fully uncovered. This conformation is close to what has been described as the “Myosin or M state” and has previously been associated with conditions in which the thin filament is fully or partially decorated with myosin heads (Behrmann et al., 2012). It has not been described previously as present in an undecorated filament.

Most previous thin filament structural analysis used either helical reconstruction techniques where tropomyosin and troponin density is averaged over each of the actin subunits (Lehman et al., 1995, Vibert et al., 1997, Xu et al., 1999) or a non-helical referenced based single particle-based analysis in which an initial model of the thin filament is used as the starting point for three-dimensional analysis (Pirani et al., 2005, Pirani et al., 2006, Yang et al., 2014). The reference-free approach used in the current analysis avoids potential reference bias and yields a significantly different and more complete density distribution for troponin than in the reference-based analysis (Pirani et al., 2005), as well as identifying differential tropomyosin conformations in distinct pseudo-repeats of the Ca^2+^-treated thin filament.

Docking of crystal structures of the troponin core complex allowed us to confirm the polarity of troponin with TnT1 located towards the pointed end of the filament. Two regions of additional density within the thin filament reconstructions, which are not present in available crystal structures (Takeda et al., 2003, Vinogradova et al., 2005), were identified. These regions can be assigned to TnT1 and the C terminus of TnI. Both of these regions are known to form important interfaces with actin and tropomyosin (Tripet et al., 1997). TnT1, the N terminal of TnT, contains a major tropomyosin binding domain (residues 114–138) and is positioned adjacent to tropomyosin in our maps, thus confirming the polarity of troponin. At elevated Ca^2+^ concentration TnT1 is docked so that it is angled across the outer surface of the tropomyosin coiled-coil (Figs. 2C, 3B–D), while at low Ca^2+^ it appears more closely integrated with tropomyosin, running parallel to the coiled-coil where it may contribute to the positioning of tropomyosin in the blocked state (Figs. 2F, 3F–H). At low Ca^2+^ concentrations the C terminus of TnI, including residues 176–184, an area homologous with the second actin-tropomyosin binding site, wraps across tropomyosin and appears to interact with the adjacent actin subunit. Prior to activation, the troponin complex appears to be held in a compact state with its interactions keeping tropomyosin in the blocked position. We can hypothesise that as Ca^2+^ binds to the N-terminal lobe of TnC, the central helix linking the two TnC lobes becomes ordered and the tropomyosin strand is effectively pushed across the face of the actin subunit (from the outer domain to the inner domain). The second actin binding site of TnI is thereby released and the C-terminus of TnI recoils away from actin.

**Fig. 5:**
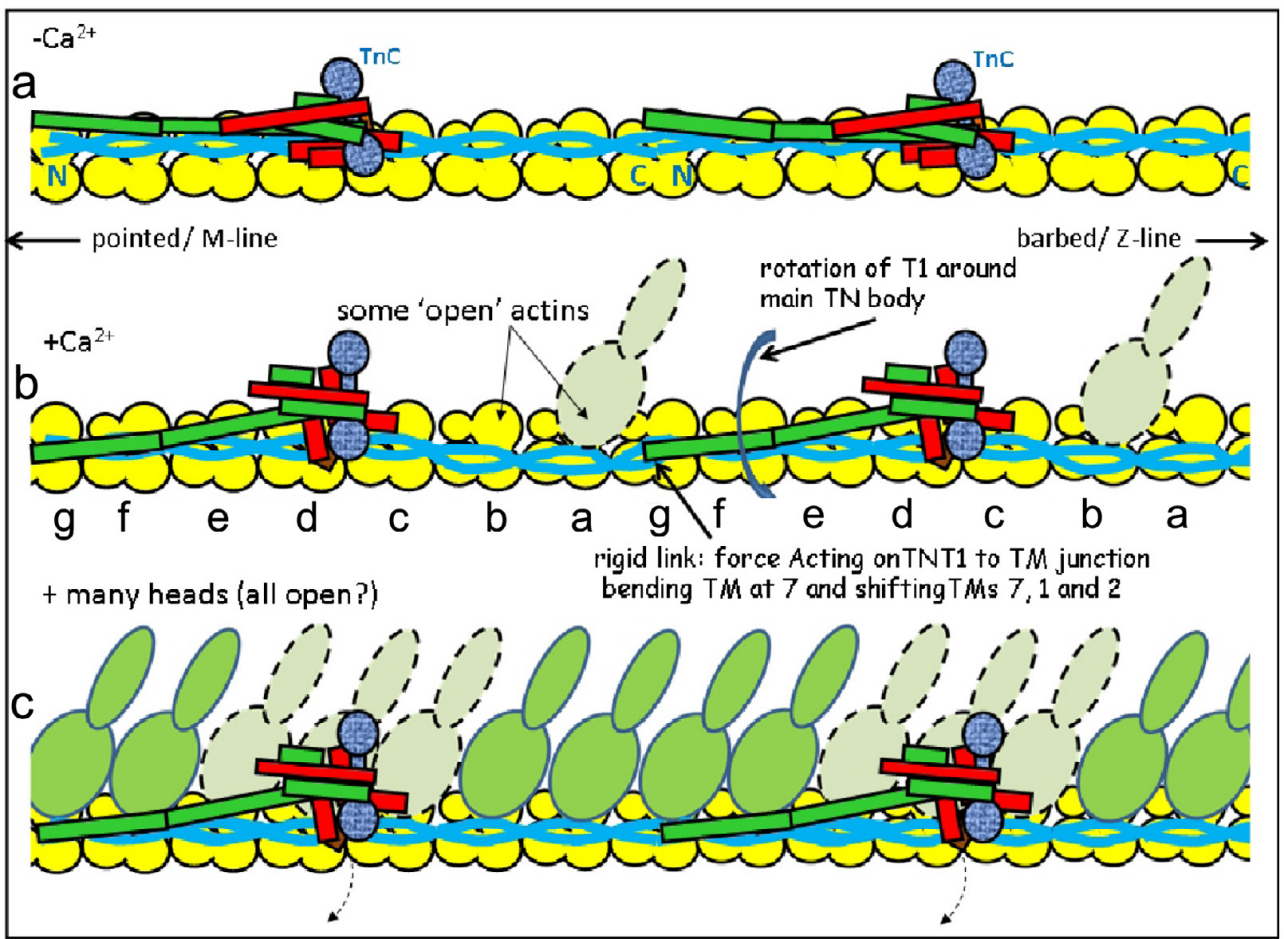
A novel mechanism of regulation. Schematic diagrams of the thin filament assembly for simplicity shown as a linear array (i.e. not helical). Protein components colour coded: (yellow), tropomyosin (blue) and troponin (TnC, speckled blue; TnI, red; TnT green). When the thin filament changes from the Ca^2+^ free state (a) to the Ca^2+^ bound state (b) the tropomyosin strands on actins g, a and b move further than those on actins c-f. Actins g, a and b may be exposed enough for some myosin heads to bind (dashed outline). Bound heads going over to strong states may then activate the whole filament (c). The body of troponin may move out of the way (dashed arrows) to permit binding of further heads. (a and b) also illustrate a potential mechanism whereby the distal arm of TnT1 acting on the tropomyosin overlap region may swing to expose the binding sites on actins g, a and b when TnC binds Ca ^2+^.

Our observations suggest that the changes in the troponin complex associated with Ca^2+^ binding lead to differential movements of the tropomyosin strands depending upon their precise location with respect to the troponin complex. In addition the seven repeats within the tropomyosin molecule are themselves only pseudo-repeats which may cause significant local differences in their actin-binding properties. The resulting positions of the tropomyosin strands at elevated Ca^2+^ range from the closed state for the repeats adjacent to actin subunits d, e, f and g on the pointed side of the troponin complex, to the apparently fully activated M-state for the repeats adjacent to actin subunits a, b and c on the barbed side of the troponin complex (Fig. 5). Although the tropomyosin strands adjacent to actin subunit c where the troponin core is located appear to correspond to the fully activated M-state, the troponin complex itself can be seen to overlap much of the myosin binding surface of actin suggesting that these subunits would be effectively closed or blocked. The coexistence of structurally defined closed and M-states in the Ca^2+^-treated thin filament has to our knowledge not previously been suggested and has significant implications for the mechanism of thin filament regulation

## 5 A structural basis for regulation

The mechanism by which the thin filament is currently thought to be regulated involves three states which differ in the position of tropomyosin: the blocked state associated with the Ca^2+^-free conformation, the closed state associated with the Ca^2+^-bound conformation and the fully active M-state which is induced by myosin binding (Lehman et al., 1995, McKillop and Geeves, 1993, Xu et al., 1999). It is assumed in this type of mechanism that individual actin subunits within a regulatory unit are in equivalent states, with dynamic variations in conformation of the whole regulatory unit (Pirani et al., 2005). Here we suggest, that the Ca^2+^-treated state of the thin filament is characterised by the coexistence of actin subunits with closed and M-state tropomyosin conformations in a distribution governed by their spatial relationship to the troponin complex (Fig. 5). The coexistence of closed and M-state tropomyosin conformations suggests a mechanism for the cooperative transition from the closed to the M state involving the initial binding of myosin to specific target actin subunits (such as a and b), then inducing activation of other parts of the same regulatory unit. Strong myosin binding to the initial target subunits may favour transition to the M-state in adjacent actin subunits and thereby the progressive transition of the thin filament into a fully activated state.

## Funding

This work was supported by the British Heart Foundation Project grant (#23480) to EPM and JMS and Career re-entry Fellowship FS/14/18/3071 awarded to DP.
